# Assessment of dentin mineral density of human teeth using micro-computed tomography in two kilovoltage levels

**DOI:** 10.1007/s10266-023-00801-4

**Published:** 2023-03-30

**Authors:** Khalid Al Hezaimi, Yaara Berdan, Ilan Rotstein

**Affiliations:** 1 Department of Endodontics and Periodontics, University of Southern California, Los Angeles, CA USA; 2 Herman Ostrow School of Dentistry of USC, University of Southern California, 925 W. 34Th Street, DEN 310, Los Angeles, CA 90089-0641 USA

## Abstract

A significant advancement in micro-computed tomography (μCT) translational application in endodontics has occurred. The purpose of the study was to assess the applications of a new method to measure dentin mineral density (DMD) and to compare between 2 levels of energy sources. Two sets of standardized porous solid hydroxyapatite (HA) phantoms, with mineral densities of 0.25 g/cm^3^ and 0.75 g/cm3, respectively were embedded in aluminum foil. The μCT homogeneity and noise in the HA phantoms were analyzed using 50 kV and 100 kV energy sources. DMD of 66 extracted human teeth was measured at the cemento-enamel junction (CEJ), mid-root, and apical levels. Assessment included linearity between the energy source and the DMD measurement. The quality of the images obtained from the 2 energy sources was compared and analyzed statistically. HA phantom rods and validation methods showed that 100 kV provided a more accurate measurement of the DMD in all groups tested. The 100 kV 3D reconstructed µCT images displayed a more defined details of the dentin structure. A statistically significant difference was found between 100 and 50 kV (*p* < 0.05) in all measured areas except for the mid-root. Using micro-computed tomography is a practical and non-destructive method to measure dentin density. 100 kV energy source provides clearer and more consistent images.

## Introduction

Micro-computed tomography (μCT) is a three-dimensional non-invasive, non-destructive imaging technique for material testing [1]. It provides a detailed assessment of an object’s internal and external structures and generates a three-dimensional (3D) reconstruction [2, 3]. μCT has a wide array of applications in dentistry that include, among others, hard tissues microarchitecture, density, and composition [4], demineralization–remineralization of dental caries [4], bone-to-implant contact [5–7], thickness and volume of calcified tissues [6], amount of hydroxyapatite (HA) in mineralized tissues, and quantitative bone structure and bone mineral density (BMD) [7–10].μCT resolution is determined by the voxel size and is mainly dependent on the scanning parameters and the characteristics of the scanned object or tissue [11]. When scanning a tooth, the region of interest (ROI) is initially defined so that the threshold includes all relevant dental structures [7]. Once scanning metrics have been defined, binarization is formed to separate the voxels representing the specific tissue from adjacent tissues and surrounding structures.

Dentin mineral density (DMD) is reflected by X-rays attenuation, which has a linear proportional relationship. This technique can measure the dentin’s calcified tissue mineral density [12]. However, μCT requires a constant and consistent calibration, which is essential for accurately reproduced measurements [1].

Measuring the tooth microarchitecture, density, and HA content provides useful information regarding the calcified tissue mineral phase. It has been reported that HA phantom calibration resulted in consistent and homogeneous outcomes [1]. Older reports described limitations of HA phantom when detecting density below 1.4 g/cm^3^ due to manufactural specifications [13, 14]. However, with advancement of μCT technology, HA phantom has demonstrated the ability to also measure lower density ranging from 0.07/cm^3^ to 1.05 g/cm^3^.

Dentin density can be determined using μCT based on spatial distribution maps of linear attenuation coefficients and determined by X-ray source’s energy and the scanned material's atomic composition [15]. This method has been used to assess demineralization and remineralization of calcified tissues. The imaging process is non-destructive, and the internal features of the sample can be examined multiple times [16]. This provides a major advantage to the examiner. To date, μCT studies examining DMD of human roots, at different levels, are scarce.

The purpose of the study was to assess the applications of a new method to measure dentin mineral density (DMD) and to compare between 2 levels of energy sources.

## Materials and methods

The study was approved by the Growth Factors and Bone Regeneration (GFBR) research board and complied with its research ethics and guidelines. A total of 66 freshly extracted human teeth were stored in a medium of 0.1% thymol at a pH of 9.1. All teeth were visually inspected using magnification and scanned radiographically. Exclusion criteria were: (1) presence of canal calcifications; (2) coronal and/or radicular caries; (3) root fracture; (4) root resorption; and (5) presence of root canal filling. The teeth were randomly divided into the following groups: Group 1- maxillary molars; Group 2- maxillary premolars; Group 3- maxillary incisors; Group 4- mandibular molars; Group 5- mandibular premolars; and Group 6- mandibular incisors. The teeth were then fixed in 10% phosphate-buffered formaldehyde (pH 7.4) and dehydrated in 70% ethanol. μCT examination was done using a scanner (Sky Scan, model 1172, Brussels, Belgium) to assess the mineral density of the dentin. Prior to μCT scanning, the cementum was removed from all teeth using hand curettes (Hu-Friedy, Chicago, IL). Each tooth was then wrapped in Parafilm (Pechiney Plastic Packaging Co, Chicago, IL) to prevent desiccation during scanning.

### Beam-hardening and correction scheme

Three-dimensional reconstruction was done using Insta Recon Software and Bruker SkyScan (Skyscan, Skyscan, Kontich, Belgium). A 30% beam hardening effect reduction and 12% ring artifact correction were used to produce a detailed image cross section. For the reconstruction, the upper and lower threshold values for dentin were determined to be 225 and 550, respectively, out of a total range of 0–1000 (0 and 1000 correspond to linear attenuation coefficients of 0–1 cm and 8.0–1 cm Hounsfield Units (HU), respectively.

### DMD calibration

**Fig. 1:**
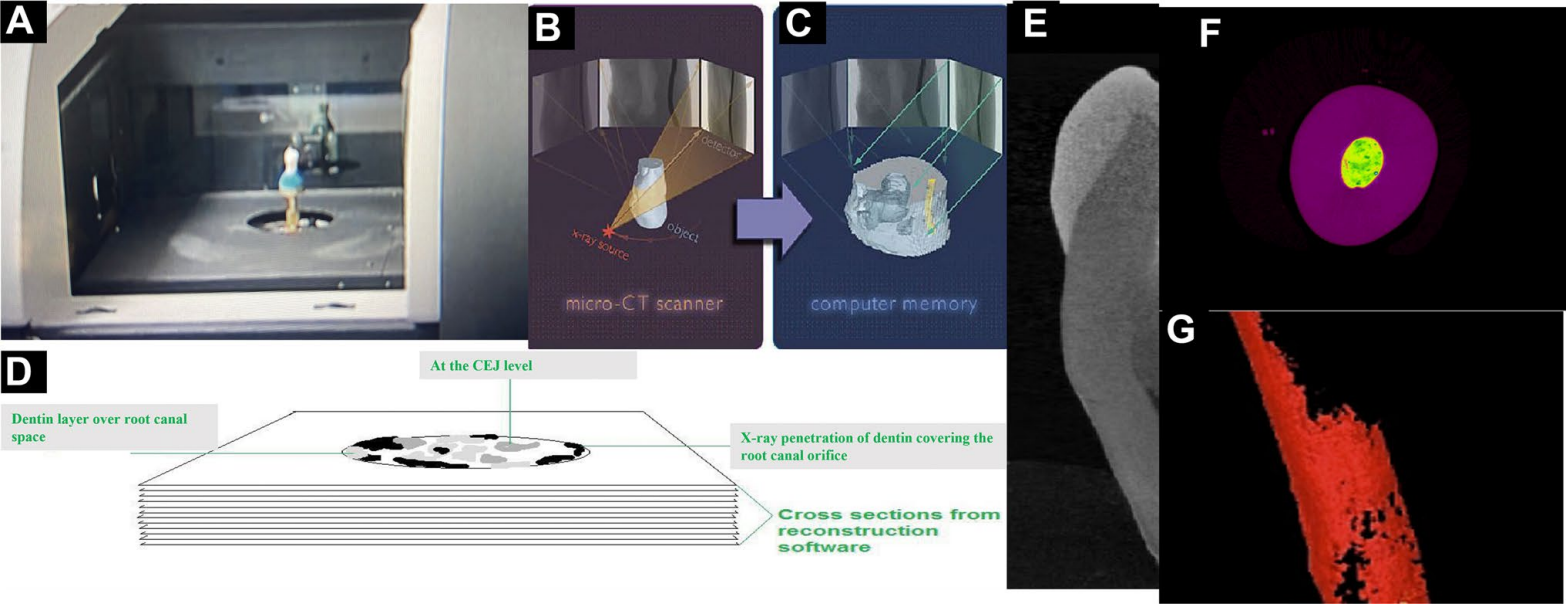
**A** Tooth in µCT machine with a stable mounting. **B** Illustration of X-ray generation from the source toward the object. **C** Illustration of data translation to computer using InstaRecon. **D** Axial cross-sectional images of X-ray data translation to the computer. **E** Sagittal section of a natural tooth. The ROI is indicated with a yellow circle at the CEJ level. **F** Axial section of the ROI perimeter borders. **G** 100 kV high-resolution axial section of 0.018 micron of the ROI showing the architectural structure of the dentin at the CEJ

**Fig. 2:**
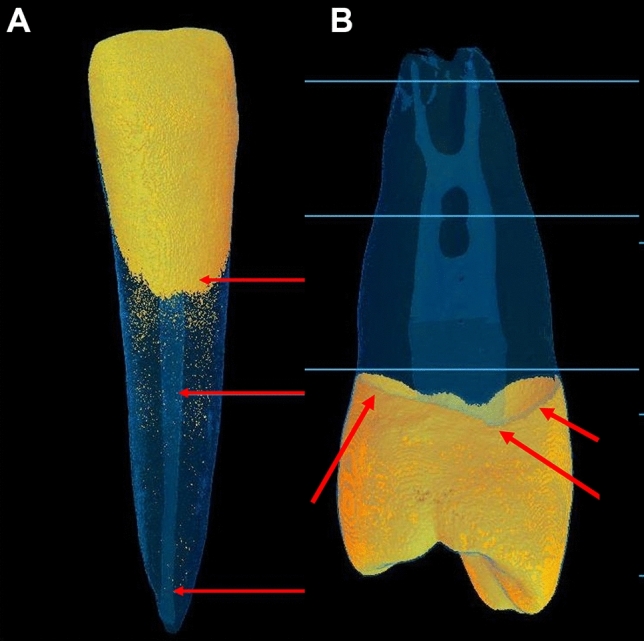
Bucco-lingual (**A**) and mesio-distal (**B**) Views of reconstructed µCT image samples of the teeth tested. **A** Arrows pointing to the CEJ, mid-root and apical area. **B** An uneven outline of the CEJ is apparent, which defines the border of the ROI at the CEJ level (arrows)

An 8 mm rod of two HA phantoms (Computerized Imaging Reference System Inc, Norfolk, VA) of density of 0.25 g/mm^3^ and 0.75 g/mm^3^ was embedded aluminum foil. HU for HA phantom calibration was done per the manufacturer's instructions [17] (Fig. 1A). Beam-hardening procedure was performed using NRecon software (NRecon, Skyscan, Kontich, Belgium) with a high-resolution desktop micro-CT scanner (Skyscan, Kontich, Belgium) to reduce voltage inconsistency (Fig. 1B and C). The selected region of interest (ROI) in the examined dentin was calibrated (Fig. 1E–G), and DMD was measured in g/mm^3^. A cylindrical ROI with a diameter of 321 pixels was examined, corresponding to 4.8 mm. One-hundred binarized slices at the cemento-enamel junction (CEJ) level, mid-root, and apical area with exact threshold values for three-dimensional (3D) analysis were measured and analyzed using the μCT analysis software (2.1.0.0) (Skyscan, Kontich, Belgium) (Fig. 2).

**Fig. 3:**
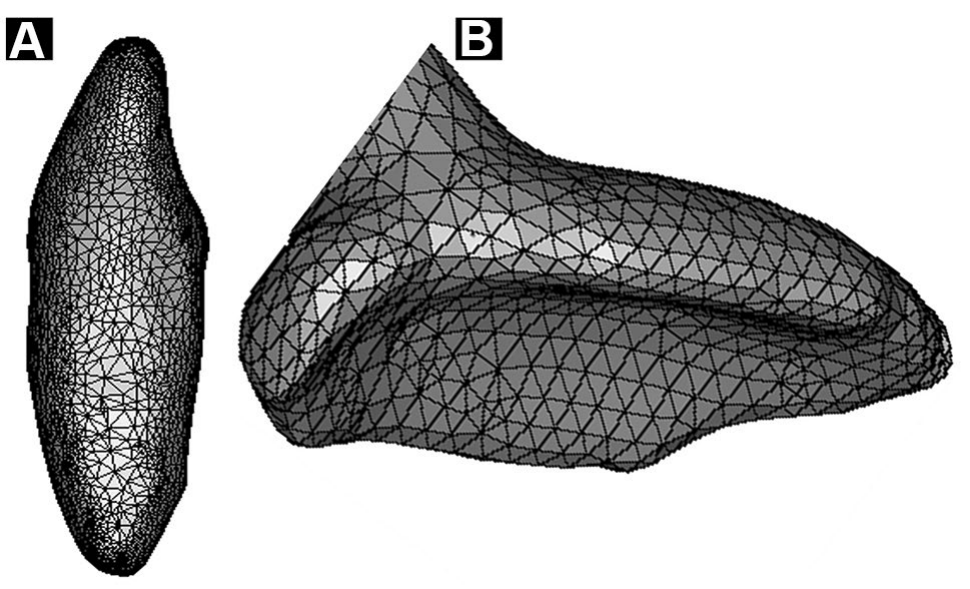
**A** 3D reconstructed µCT image sample of an anterior tooth used to define the ROI. **B** A reconstructed image sample used to define the ROI at the CEJ level. Binarization to define the ROI is used to measure its DMD

**Fig. 4:**
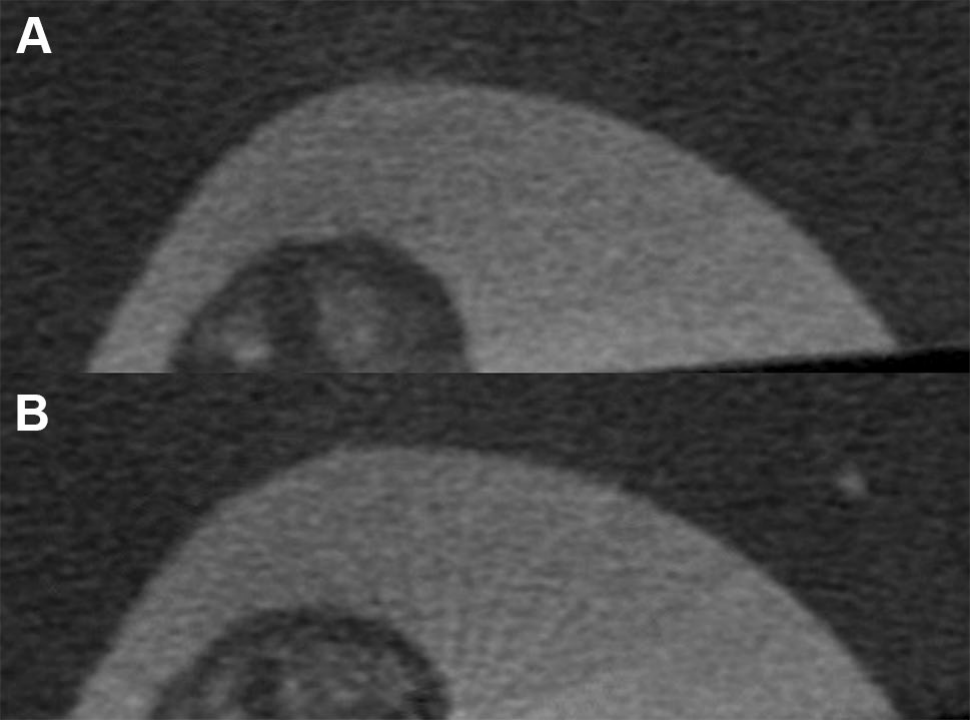
3D reconstructed µCT images of the ROI area shown in Fig. 1E using the two energy sources studied. **A** 50 kV image shows a vaguer appearance of the dentin structures and a less defined architecture. **B** 100 kV image shows a more defined appearance of the dentin structures and trabeculation

ROI for DMD was defined at the CEJ, mid-root, and apical third (Fig. 3). A 32-frame averaging was applied in the acquisition phase, three-frame averaging mode, random movement mode, geometrical correction, and flat-field correction were made to improve the signal-to-noise ratio. A new flat-field reference was applied to reduce ring artifacts before scanning. The X-ray generator of the μCT was operated at an accelerated potential of 50 kV or 100 kV with a beam current of 98 microamperes (mA) using an aluminum filter with a pixel resolution of 37.41 µm. The rotation step was set at 0.7°, resulting in 272 two-dimensional projections over a 180° rotation of the sample tested. Exposure time was 3000 microseconds (ms). All images were taken at a voxel size of 33 × 33 × 33 µm^3^. The greyscale definition is proportionally related to the quality of the X-ray penetration of the structural object (Fig. 4). The DMD was calculated using the following formula: Density = Mass (weight)/volume. The accuracy of the calibration method for the two energy sources examined was assessed by inspecting the consistency of each energy source's DMD result.

The accuracy of the calibration method was appraised for the two different energy levels tested. The HA phantoms were utilized to establish the calibration base for both energy sources where the measured gray value was converted to an estimated mineral density. One technician with 12 years of experience in micro-computed tomography scanning and analysis performed the scanning and measurement. Intra-calibration was performed, and the Kappa index was 0.90.

### Statistical analysis

The data obtained were manually entered into a Statistical Package for the Social Sciences database (IBM, SPSS version 20, IL, USA). One-way analysis of variance (ANOVA) was used at a 95% level of confidence to assess the statistical difference between the groups.

## Results

Calibration comparisons between the 2 rods HA phantom using 50 kV and 100 kV are shown in Table 1. The mean of DMD when using 50 kV for groups 1, 2, 3, 4, 5, and 6 was 4.05, 4.39, 5.79, 4.14, 4.89, and 5.57, respectively. There was no significant difference between Groups 1 and 4, Groups 2 and 5, and Groups 3 and 6. The mean of DMD when using 100 kV for groups 1, 2, 3, 4, 5, and 6 was 5.92, 5.45, 7.69, 6.11, 5.62, and 6.48, respectively. A non-significant difference was found between groups 1 and 4, groups 2 and 5, and groups 3 and 6. μCT operating at 100 kV was found to grant more accurate measurement of DMD in all tooth groups tested (Tables 2, 3, 4). In molars, it was statistically significant at the CEJ and apical levels (Table 2). At the mid-root level, 100 kV yielded higher accuracy than 50 kV; however, the difference was not statistically significant. In premolars, 100 kV yielded significantly higher DMD accuracy than 50 kV in all levels of the root (Table 3). In anterior teeth, 100 kV was also significantly more accurate in all levels of the root (Table 4). 3D reconstructed 50 kV µCT images showed a slightly vague radiopaque dentinal structure, and less-defined dentin architecture (Fig. 4). 3D reconstructed 100 kV µCT images produced a more defined dentin structure and trabeculation (Fig. 4).

**Table 1:** Calibration comparison of 0.25 g/cm^3^ (A) and 0.75 g/cm^3^ (B) rods HA phantom using the two energy levels studied

	A (mean ± SD)	B (mean ± SD)
50 kV	1190.86 ± 0.9	3591.98 ± 1.41
100 kV	1203.98 ± 0.15	3642.07 ± 0.03

*SD* standard deviation

**Table 2:** Dentin mineral density of molar roots using two kilovoltage (kV) levels

	50 kV	100 kV
	Mean ± SD	Mean ± SD
CEJ	4.09 ± 2.47	6.01 ± 0.46*
Mid-root	5.96 ± 1.43	6.38 ± 0.43
Apex	7.43 ± 1.60	8.99 ± 0.53*

*CEJ* cemento-enamel junction, *Apex* apical third, *SD* standard

^*^p < 0.01

**Table 3:** Dentin mineral density of premolar roots using two kilovoltage (kV) levels

	50 kV	100 kV
	Mean ± SD	Mean ± SD
CEJ	4.64 ± 2.04	5.53 ± 0.51*
Mid-root	4.74 ± 1.2	5.58 ± 0.49*
Apex	5.95 ± 2.15	7.80 ± 0.41**

*CEJ* cemento-enamel junction, *Apex* apical third, *SD* standard deviation

^*^p < 0.01

^**^p < 0.05

**Table 4:** Dentin mineral density of anterior roots using two kilovoltage (kV) levels

	50 kV	100 kV
	Mean ± SD	Mean ± SD
CEJ	5.66 ± 2.74	7.01 ± 0.77*
Mid-root	5.95 ± 2.75	7.46 ± 0.35*
Apex	2.27 ± 0.69	7.72 ± 0.56**

*CEJ* cemento-enamel junction, *Apex* apical third, *SD* standard deviation

^*^p < 0.01

^**^p < 0.001

## Discussion

The current study confirms that energy source impacts the measurement of a gray value-to-mineral density of dentin. Also, there was a significant difference in each DMD measurement when dentin was scanned in 100 kV vs. 50 kV. Our results are in agreement with the previous report [18]. When 50 kV was used to scan HA phantom rods, it produced a significant lower image quality than that of the same PH rods scanned at 100 kV. Therefore, μCT scanning at 100 kV produced more consistent measurements, better image quality, less signal-to-noise ratio, and well-defined details of the scanned object [18]. Our study is also in alignment with previous reports [19, 20] showing a linear relationship between X-ray attenuation coefficient and tissue mineral density. Indexed literature [5–8, 10–12, 16, 23, 26] pointed out that DMD measurement may differ between different operating devices [4, 19, 21–25].

A 100 kV energy source has consistent, stable, and produced more accurate density based on HA phantom calibration, and the 100 kV maintained the same accuracy and consistency when scanning natural teeth. One critical outcome when using 100 kV was structural definition, details, and dentin porosity identification was significantly better.

BMD is commonly utilized to measure bone quality and strength is used as a diagnostic tool for osteoporosis and for assessment of therapeutic results [26]. DMD can be used to measure density, thickness, and quality of human dentin as a response to stimuli or after applications of different biomaterials.μCT DMD measurement methods can be transferred to computed beam Cone-beam computed tomography systems (CBCT) as they are the same radiological imaging type. For example, various clinical applications need to measure DMD to assess treatment outcomes, such as vital pulp therapy, apexogenesis, and pulpal revitalization/regeneration. AlHezaimi et al. [2] suggested using μCT quantitative assessment of human teeth receiving vital pulp therapy.

Current radio-graphical image analysis is subjective and only shows the newly formed dentin in the mesio-distal dimension and without quantitative analysis. Also, it doesn’t provide a quantitative assessment to reflect the quality, thickness, and whether the newly formed dentin seals the root canal orifice. On the other hand, the technique studied here provides a baseline measurement for DMD of extracted human teeth also at the CEJ, which can be used as a reference for future CBCT measurements. Oral healthcare providers and investigators will recognize the advantages of using technology applications of μCT and CBCT to determine dentin density in a non-destructive manner. The current CBCT device's energy source kilovoltage ranges between 70 and 140 kV. The radiation dose, depending on the parameters of the CBCT scanning, is about 4 times greater than digital panoramic radiograph. Still, CBCT is considered safe when all proper precautions are taken.

The limitation of the study is that human extracted teeth didn’t receive a vital pulp therapy. Additionally, before any DMD measurements, the CBCT calibration using HA phantoms must be first established. Further expanded clinical studies are warranted.

## References

[1] Burghardt AJ, Kazakia GJ, Laib A, Majumdar S (2008). Quantitative assessment of bone tissue mineralization with polychromatic micro-computed tomography. Calcif Tissue Int.

[2] Al-Hezaimi K, Naghshbandi J, Alhuzaimi R, Alonizan F, Al-Qwizany I, Rotstein I (2020). Regeneration of secondary dentin using recombinant human platelet-derived growth factor and MTA for pulp capping: a randomized controlled human clinical trial. Int J Periodontics Restorative Dent.

[3] Al-Hezaimi K, Naghshbandi J, Alhuzaimi R, Alonizan F, Al-Qwizany I, Rotstein I (2020). Evaluation of recombinant human platelet-derived growth factor or enamel matrix derivative plus calcium hydroxide for pulp capping: a randomized controlled human clinical trial. Int J Periodontics Restorative Dent.

[4] Willmott NS, Wong FS, Davis GR (2007). An X-ray microtomography study on the mineral concentration of carious dentine removed during cavity preparation in deciduous molars. Caries Res.

[5] Al-Shabeeb MS, Al-Askar M, Al-Rasheed A, Babay N, Javed F, Wang HL, Al-Hezaimi K (2012). Alveolar bone remodeling around immediate implants placed in accordance with the extraction socket classification: a three-dimensional microcomputed tomography analysis. J Periodontol.

[6] Al-Hazmi BA, Al-Hamdan KS, Al-Rasheed A, Babay N, Wang HL, Al-Hezaimi K (2013). Efficacy of using PDGF and xenograft with or without collagen membrane for bone regeneration around immediate implants with induced dehiscence-type defects: a microcomputed tomographic study in dogs. J Periodontol.

[7] Al Hezaimi K, Naghshbandi J, Nooh N, Schupbach P, Nevins M (2021). Buccal bone remodeling around immediate implants in STZ-induced diabetic dogs: a histologic and microcomputed tomographic analysis. Int J Periodontics Restorative Dent.

[8] Mashiatulla M, Ross RD, Sumner DR (2017). Validation of cortical bone mineral density distribution using micro-computed tomography. Bone.

[9] Ramalingam S, Al-Rasheed A, ArRejaie A, Nooh N, Al-Kindi M, Al-Hezaimi K (2016). Guided bone regeneration in standardized calvarial defects using beta-tricalcium phosphate and collagen membrane: a real-time in vivo micro-computed tomographic experiment in rats. Odontology.

[10] Binsalah MA, Ramalingam S, Alkindi M, Nooh N, Al-Hezaimi K (2019). Guided bone regeneration of femoral segmental defects using equine bone graft: an in-vivo micro-computed tomographic study in rats. J Invest Surg.

[11] Christiansen BA (2016). Effect of micro-computed tomography voxel size and segmentation method on trabecular bone microstructure measures in mice. Bone Rep.

[12] MacNeil JA, Boyd SK (2007). Accuracy of high-resolution peripheral quantitative computed tomography for measurement of bone quality. Med Eng Phys.

[13] He LH, Standard OC, Huang TT, Latella BA, Swain MV (2008). Mechanical behaviour of porous hydroxyapatite. Acta Biomater.

[14] Goodsitt MM (1992). Conversion relations for quantitative CT bone mineral density measured with solid and liquid calibration standards. Bone Miner.

[15] Elliott JC, Wong FS, Anderson P, Davis GR, Dowker SE (1998). Determination of mineral concentration in dental enamel from X-ray attenuation measurements. Connect Tissue Res.

[16] Ramalingam S, Basudan A, Babay N, Al-Rasheed A, Nooh N, Nagshbandi J, Aldahmash A, Atteya M, Al-Hezaimi K (2016). Efficacy of mucograft vs conventional resorbable collagen membranes in guided bone regeneration around standardized calvarial defects in rats: a histologic and biomechanical assessment. Int J Periodontics Restorative Dent.

[17] SkyScan. Method note: bone mineral density (BMD) calibration in Skyscan CT-analyser. 2006.

[18] Chappard C, Basillais A, Benhamou L, Bonassie A, Brunet-Imbault B, Bonnet N, Peyrin F (2006). Comparison of synchrotron radiation and conventional x-ray microcomputed tomography for assessing trabecular bone microarchitecture of human femoral heads. Med Phys.

[19] Zou W, Gao J, Jones A, Hunter A, Swain M (2009). Characterization of a novel calibration method for mineral density determination of dentine by X-ray micro-tomography. Analyst.

[20] Dowker S, Elliott J, Davis G, Wilson R, Cloetens P (2004). Synchrotron x-ray microtomographic investigation of mineral concentrations at micrometre scale in sound and carious enamel. Caries Res.

[21] Hahn SK, Kim JW, Lee SH, Kim CC, Hahn SH, Jang KT (2004). Microcomputed tomographic assessment of chemomechanical caries removal. Caries Res.

[22] Clementino-Luedemann T, Kunzelmann K (2006). Mineral concentration of natural human teeth by a commercial micro-CT. Dent Mater J.

[23] Wong F, Elliott J, Anderson P, Davis G (1995). Mineral concentration gradients in rat femoral diaphyses measured by X-ray microtomography. Calcif Tissue Int..

[24] Schweizer S, Hattendorf B, Schneider P, Aeschlimann B, Gauckler L, Müller R, Günther D (2007). Preparation and characterization of calibration standards for bone density determination by micro-computed tomography. Analyst.

[25] Postnov A, Vinogradov A, Dyck V, Saveliev S, De Clerck N (2003). Quantitative analysis of bone mineral content by x-ray microtomography. Physiol Meas.

[26] Lenchik L, Shi R, Register TC, Beck S, Langefeld C, Carr J (2004). Measurement of trabecular bone mineral density in the thoracic spine using cardiac gated quantitative computed tomography. J Comput Assist Tomograph.

